# Social-emotional competence and German academic achievement among Chinese university students: a structural equation modeling study

**DOI:** 10.3389/fpsyg.2026.1816321

**Published:** 2026-05-22

**Authors:** Mengdi Zhao, Wei Zhuang, John W. Schwieter

**Affiliations:** 1 School of International Studies, Zhejiang University, Hangzhou, China; 2 Department of Psychology, Language Acquisition, Multilingualism, and Cognition Lab, Wilfrid Laurier University, Waterloo, ON, Canada; 3 Department of Linguistics, McMaster University, Hamilton, ON, Canada; 4 School of Education, University College Dublin, Dublin, Ireland

**Keywords:** German academic achievement, languages-other-than-English (LOTEs), mediation effects, social-emotional competence, structural equation model

## Abstract

**Introduction:**

Social-emotional competence (SEC), including self-awareness, self-management, social awareness, and social management, has been widely recognized as a critical factor in personal development, social adaptation, and effective interpersonal communication. As a multifaceted skill set, SEC supports holistic growth and is regarded as a strong predictor of academic success. Despite growing attention in educational studies, the application of SEC in research on learners of languages-other-than-English (LOTEs) remains underexplored.

**Methods:**

This study examined the associations between SEC and German academic achievement among Chinese university students majoring in German as a foreign language (GFL). A total of 247 third-year German majors from 21 universities in mainland China participated in the study. Using structural equation modeling and mediation effect estimation, we examined whether self-awareness, self-management, social awareness, and social management were associated with foreign language academic achievement.

**Results:**

Participants reported moderate-to-high levels of SEC. SEC was significantly and positively correlated with German academic achievement. Self-awareness was positively associated with German achievement both directly and indirectly through self-management, whereas social awareness showed a significant positive indirect association with German achievement through self-management only. Social management did not significantly mediate the associations between awareness-related competences and achievement.

**Discussion:**

The findings clarify how different SEC dimensions are associated with academic achievement in a Chinese GFL context. They highlight self-management as a key mediating mechanism and suggest that socially oriented competences may contribute to test-based language achievement primarily through internalized self-regulatory processes. These results extend SEC research beyond English-learning contexts and offer implications for integrating social-emotional learning into foreign language education.

## 1 Introduction

In recent years, social-emotional competence (SEC) has become a focal point within international educational research. However, since the 21st century, studies have gradually shifted from examining cognitive abilities (basic skills such as reading, writing, and arithmetic; knowledge memorization; and problem-solving) to non-cognitive abilities (emotion regulation, goal orientation, perseverance, and collaboration). Recently, SEC has been referred to as a “core 21st-century competency” (Huang et al., 2022). The Collaborative for Academic, Social, and Emotional Learning (CASEL) defines social-emotional learning as the ability to manage one's emotions, build positive relationships, and make responsible decisions in social interactions (Durlak et al., 2015). Empirical evidence demonstrates that SEC is positively associated with academic achievement (Borghans et al., 2008; Duckworth and Seligman, 2005; Durlak et al., 2011). Despite growing scholarly interest in SEC across education, psychology, and sociology, little research predominantly examines preschool and K-12 learners, leaving undergraduate learners underexamined. Universities play a central role in developing both high-level professionals and innovative talent, and their undergraduate studies constitute a critical period for learners' social development. Thus, the systematic cultivation of SEC at this level is imperative. Furthermore, investigations into SEC among foreign language majors—particularly learners of languages-other-than-English (LOTEs)—are rare. Language learning, distinct from other subject learning, could be emotionally sensitive and socially dependent (Bai et al., 2024).

In the present study, we examine how four dimensions of SEC, namely self-awareness, self-management, social awareness, and social management, are associated with foreign language academic achievement among Chinese students majoring in German as a foreign language (GFL). We anticipate that the findings will offer practical insights and a data-driven foundation for integrating social-emotional learning into pedagogical approaches and curriculum design to holistically support foreign language learners' linguistic, cognitive, and affective development.

## 2 Background

### 2.1 Conceptual framework and core dimensions of social-emotional competence

Research on social-emotional competence began in the early 20th century. Perhaps the earliest work was by American psychologist Edward Thorndike (1920), who introduced the concept of *social intelligence*, defined as the ability to perceive the value of social relationships, adopt others' perspectives, and build satisfying relationships. Despite its early introduction, social intelligence remained underdefined for decades. It was not until Salovey and Mayer (1990) extended Thorndike's ideas that the term *emotional intelligence* was coined, referring to the capacity to monitor one's own and others' feelings, distinguish among them, and use that information to guide thoughts and behaviors. Building on these ideas, Goleman (2005) proposed a framework of emotional intelligence, identifying five core domains: Self-awareness, self-regulation, motivation, empathy, and social skills.

Although these early studies were somewhat vague, they shared a common ground that laid the foundation for later elaborations of SEC. Founded in 1994, the Collaborative for Academic, Social, and Emotional Learning (CASEL) has played an important role in advancing the integrated development of social-emotional learning. CASEL (2012) defines SEC as the process by which young individuals acquire and apply their knowledge, skills, and attitudes to develop healthy identities, manage emotions, achieve personal and collective goals, feel and show empathy for others, establish and maintain supportive relationships, and make responsible and caring decisions. This definition has motivated scholars to conduct in-depth explorations of the dimensions of SEC. Drawing on the social-emotional learning framework proposed by the Anchorage School District, Cefai and Cavioni (2014) refined a two-dimensional, four-domain model of social-emotional learning:

(1) **Self-awareness**: recognizing one's emotions, describing personal interests and values, accurately assessing strengths, reflecting on thoughts and learning processes with confidence, self-efficacy, and autonomy;
(2) **Self-management**: setting and monitoring personal and academic goals, engaging in positive talk, controlling impulses and persevering in overcoming obstacles, solving problems effectively, and making informed decisions;
(3) **Social awareness**: taking the perspective of and empathizing with others, recognizing and appreciating individual and group similarities and differences, diversity and social inclusion; and
(4) **Social management**: possessing interpersonal skills to establish and maintain cooperative, beneficial relationships, managing and resolving conflicts, seeking and offering help when needed, and demonstrating ethical behavior and responsible decision-making.

Cefai and Cavioni's (2014) social-emotional learning model synthesizes the fundamental competencies identified by CASEL—self-awareness, self-management, social awareness, relationship skills, and responsible decision-making (Weissberg et al., 2015). Crucially, the model extends beyond the individual level to integrate a societal dimension, countering the individualistic orientation prevalent in Western contexts that often marginalize collective responsibility (Cefai and Cavioni, 2014).

### 2.2 Social-emotional competence and learning achievements

Empirical studies consistently demonstrate that SEC is a principal predictor of students' academic achievement across diverse contexts. Duckworth and Seligman (2005) found that students with high self-discipline, ambitious academic goals, and strong emotion management tend to have better achievements. Additionally, Durlak et al. (2011) conducted a meta-analysis of 213 school-based social-emotional learning programs and reported significant improvements in participants' academic indicators. Zins et al. (2004) used numerous empirical cases to show that social-emotional learning plays a key role in raising children's academic achievement and promoting lifelong learning. In a large-scale survey of primary students in western China, Chen and Mao (2016) identified SEC as a primary mediator between family capital and scholastic success, indicating that socioeconomic advantages are transmitted to academic outcomes via emotional and interpersonal skills. Additionally, recent findings have shown that SEC predicts academic attainment through pathways that mediate academic emotions, learning strategies, and teacher-student relationships (Liu et al., 2022). Taken together, these empirical findings indicate that SEC is a foundational contributor to learning achievement, elucidating our understanding of the complex psychological and relational processes through which non-cognitive skills shape cognitive outcomes.

### 2.3 Social-emotional competence and foreign language academic achievement

Despite the growing attention to the relationship between SEC and academic achievement, empirical research on the link between SEC and foreign-language achievement remains limited. Bai et al. (2024) examined the complex relationship between SEC and English academic achievement among secondary students in Hong Kong and found that the four aforementioned SEC dimensions showed significant positive associations with English achievement. The study provides an important basis for understanding SEC in language learning. This framework has subsequently been extended to Hong Kong primary school learners, where self-management emerged as the proximal mediator linking motivational beliefs to English achievement, with social management failing to predict performance directly (Shen et al., 2024). At the university level, Zhang et al. (2026) similarly confirmed that self-management functioned as the sole independent mediator between academic buoyancy and English achievement among Chinese EFL undergraduates, with awareness skills operating upstream through serial mediation. These findings suggest that the awareness-to-management pathway may generalize across educational levels in Chinese learning contexts, a possibility the present study examines in the underexplored domain of L3 German learning. At the same time, the majority of relevant research in applied linguistics has been conducted within English as a Foreign Language (EFL) contexts, predominantly focusing on individual affective variables such as enjoyment, anxiety, or grit (Dewaele and MacIntyre, 2014; Li and Wei, 2023; Li and Han, 2022; Saito et al., 2018; Teimouri et al., 2019; Zhao and Wang, 2023), rather than on the coordinated operation of multiple social-emotional competences. This limitation becomes particularly salient in the case of learners of LOTEs, whose learning conditions often differ substantially from those reported on in mainstream EFL research.

The rapid expansion of non-English foreign language programs in Chinese universities, driven in part by national strategic initiatives such as the Belt and Road policy, has created an urgent demand for learners proficient in LOTEs (Han et al., 2019; Lu and Shen, 2022). Yet, this expansion has also introduced distinctive challenges. Most students entering German programs in China have prior formal foreign language experience almost exclusively in English and are expected to meet demanding academic standards within a relatively compressed timeframe. For example, German majors in mainland China are required to take the national standardized Prüfung für das Germanistik-Grundstudium (PGG, Test for German Majors 4) by the end of their second academic year. As a high-stakes assessment taken at a relatively early stage of learning, the PGG places considerable pressure on students. Under such structural and institutional conditions, the affective and regulatory dimensions of learning become especially salient.

A further issue, that has received insufficient attention, is that for most Chinese university students majoring in German, German is not a second language (L2) but a third language (L3), learned after Chinese (L1) and English (L2). Research on L3 acquisition has consistently shown that L3 learning may differ qualitatively from L2 learning because multilingual learners draw on a more complex linguistic repertoire. Previously acquired languages may both facilitate and constrain development through cross-linguistic influence, transfer, heightened metalinguistic awareness, and competition among languages (Cenoz, 2013; Cenoz et al., 2001; De Angelis, 2007; Jessner, 2008). For Chinese GFL learners, German acquisition is therefore shaped not only by the target language itself, but also by the continued influence of English and the need to manage multiple non-native language systems.

This L3 perspective also has important affective implications. On the one hand, prior experience with English may provide multilingual learners with greater language-learning experience, more developed strategies, and stronger metalinguistic resources, which may offer cognitive support in some aspects of additional language learning (Cenoz, 2013). On the other hand, multilingual learning may also increase emotional demands because learners must manage interference across languages, ambiguity arising from competing linguistic systems, and pressure associated with learning another foreign language within a limited timeframe. Research suggests that the relationship between multilingualism and foreign language anxiety is not uniform, and that affective outcomes are shaped by a range of individual and contextual factors (Dewaele, 2007; Dewaele et al., 2008, 2024; Thompson and Lee, 2013). For Chinese GFL students, the emotional experience of learning German may therefore differ in meaningful ways from that of learners studying English as their only foreign language.

This distinction matters for the present study because it helps explain why the SEC-achievement relationship may be partly similar to, yet not identical with, the pattern reported in English-learning contexts. The relationship may be similar in that successful language learning in both EFL and GFL settings requires learners to regulate emotions, sustain motivation, and mobilize social support, all of which fall within the scope of SEC. At the same time, the relationship may differ because German-major students in China are navigating a more demanding multilingual learning ecology. In such a context, the demands placed on both self-related and socially oriented competences may differ from those documented in EFL settings, though the precise nature of these differences is an empirical question. The L3 configuration of Chinese GFL learners thus provides a theoretically motivated basis for extending SEC research beyond EFL contexts: it allows us to examine whether the structural relationships among SEC dimensions and academic achievement identified in English-learning settings remain stable, or are reconfigured, when the target language is learned as an L3 within a high-pressure university environment.

## 3 Present study

Existing research on the link between SEC and foreign language achievement appears to have three key limitations. First, although SEC has been shown to benefit students' academic achievement, the internal interplay of its dimensions, i.e., self-awareness, self-management, social awareness, and social management, remains underexplored. Prior studies have treated SEC as a single, unified construct, overlooking how its separate competence dimensions interact in relation to students' academic achievement. Second, prior work has focused mostly on adolescent learners, leaving undergraduate students—whose social and emotional skills differ substantially—largely overlooked. Third, few investigations have examined these issues among students of LOTEs, and thus, any potential effects among this population are unclear. To address these gaps, the present study samples third-year GFL students at Chinese universities, and operationalizes SEC as a unified theoretical construct to investigate its associations with German academic achievement and to assess how SEC dimensions interact.

### 3.1 Theoretical model and hypotheses

Bai et al. (2024) built on Cefai and Cavioni's (2014) social-emotional learning framework by proposing a structural equation model with mediation effects to examine the relationships among four core SEC dimensions and English academic achievement. Their model was informed by Self-Determination Theory, which holds that satisfaction of learners' basic psychological needs for autonomy, competence, and relatedness supports motivation, emotional well-being, and adaptive behavior (Deci and Ryan, 1985; Ryan and Deci, 2000). In line with this perspective, Bai et al. (2024) proposed that awareness-related competences would predict English learning achievement both directly and indirectly through management-related competences. Their SEM analyses ultimately confirmed two mediation paths, highlighting the mediating role of self-management in linking both self-awareness and social awareness to English learning achievement.

The present study builds on Bai et al.'s framework while extending it to Chinese university students majoring in German. This extension is theoretically necessary because Chinese GFL learning takes place in a distinct multilingual and institutional context. As discussed in Section 2.3, for Chinese learners majoring in German, it is most typical that the target language (German) is their L3, learning it after their other two languages (Chinese and English). This situation places learners within a more complex multilingual learning environment in which they must manage cross-linguistic demands, navigate the affective challenges associated with additional language learning, and meet demanding academic standards within a compressed timeframe. This context raises the question of whether the structural pathways identified by Bai et al. (2024) in an EFL setting will be replicated among Chinese GFL learners, and whether the relative contributions of self-related and socially oriented SEC dimensions may differ given these distinct learning conditions. Recent evidence from comparable Chinese university contexts, in which self-management has emerged as the key mediating mechanism linking psychological resources to language achievement (Zhang et al., 2026), lends partial support to such replication while leaving open the extent to which these patterns hold in a high-stakes L3 setting.

Two additional theoretical perspectives inform the hypotheses of the present study. First, control-value theory offers a complementary lens for understanding why awareness-related competences may be linked to achievement partly through management-related competences (Pekrun, 2006). According to this theory, learners' appraisals of control and value shape their emotions of achievement, which in turn influence engagement, strategy use, and performance. Within this framework, learners who are more aware of their emotional states, strengths, and goals may be better positioned to mobilize the self-regulatory strategies that sustain academic performance. We therefore predict that self-management will mediate the association between self-awareness and German academic achievement. Second, sociocultural theory (Vygotsky, 1978) informs expectations about the role of socially oriented SEC dimensions. This perspective holds that learning is mediated through social interaction and culturally organized activity, and that externally available social resources may be internalized and transformed into individual regulation over time. In the present GFL context, where learning is assessed through a high-stakes, individually administered written examination, socially oriented competences may contribute to achievement primarily through internalized self-regulatory processes rather than through direct behavioral pathways. Accordingly, social awareness and social management are expected to be positively associated with German language achievement, with their effects being expressed indirectly through self-management rather than through direct pathways.

**Figure 1:**
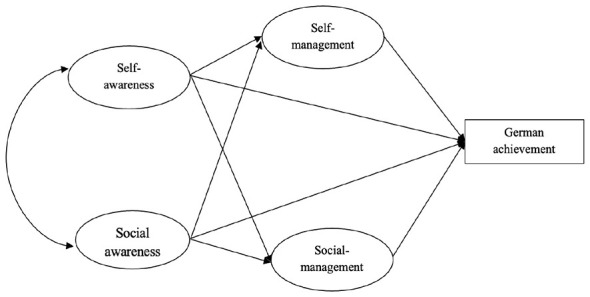
Hypothesized model of social-emotional competence and German achievement, conceptually adapted from Bai et al. (2024).

Against this background, the present study adapts Bai et al.'s (2024) model to examine Chinese GFL students and ask whether: (1) self-awareness and social awareness are positively associated with German achievement; and whether (2) self-management and social management mediate the associations between awareness-related competences and German achievement. As shown in Figure 1, we hypothesize that self-awareness and social awareness will show positive associations with German academic achievement, both directly and indirectly through self-management and social management. Building on the theoretical perspectives outlined above, we further predict that the contribution of socially oriented competences to German achievement will be expressed primarily through indirect pathways via self-management, rather than through direct associations—given that the high-stakes, individually administered nature of the PGG places a premium on individual, self-regulatory performance instead of on collaborative engagement. Overall, the four dimensions of social-emotional competence are expected to be jointly associated with German academic achievement, though the relative salience of direct versus indirect pathways may differ from the pattern reported in English-learning contexts.

### 3.2 Research context and participants

To control for differences in grade level, we focused on third-year GFL students who had already taken the PGG. Data were collected via an online survey using convenience sampling at 21 universities in mainland China. Specifically, the 21 universities were located across 10 provinces and municipalities, including Shanghai, Jiangsu, Zhejiang, Shandong, Beijing, Guangdong, Anhui, Sichuan, Chongqing, and Shaanxi, thereby capturing variation in educational resources and sociocultural contexts. A total of 262 questionnaires were returned. After data cleaning (i.e., filtering for grade level, removing invalid responses with attention-check items, and deleting patterned or overly fast responses of less than 3 min), 247 (94.27%) valid questionnaires remained. Among the valid respondents, 21.5% were male and 78.5% were female, with ages ranging from 20 to 22years (*M* = 20.80, *SD* = 0.63). In terms of institutional background, 52.38%of participants were from “Double First-Class” universities, 42.86%from regular public universities, and 4.76% from independent colleges, reflecting the stratified structure of GFL programs across Chinese higher education.

### 3.3 Materials

#### 3.3.1 Social-emotional competence questionnaire for German language learning

We adopted a “Social-Emotional Competence Questionnaire for German Learning” (see the Appendix) which was based on Bai et al.'s (2024) version for English learning. The questions in the measures were based on five-point Likert scales (1 = “strongly disagree” to 5 = “strongly agree”) and encompassed 24 items across four dimensions: self-awareness, self-management, social awareness, and social management. Bai et al.'s original questionnaire demonstrated strong reliability (Cronbach's α = 0.919) and validity (structural validity: χ^2^/df = 542.659/243 < 3; CFI = 0.918 > 0.90; TLI = 0.907 > 0.90; SRMR = 0.056 < 0.08; RMSEA = 0.063 < 0.08). To ensure linguistic and conceptual equivalence, we followed a systematic back-translation procedure. The original English questionnaire was first translated into Chinese by a bilingual researcher with expertise in applied linguistics and then independently back-translated into English by a second bilingual researcher blind to the original version. Any discrepancies between the back-translated and original versions were identified and resolved through discussion between the translators and the research team. Of the 24 items, 20 showed close semantic correspondence between the back-translated and original versions and required no modification; the remaining four items were revised accordingly to achieve semantic and conceptual equivalence. The contextual framing of all items was adapted from English learning to German learning (e.g., “I know my strengths and weaknesses in German learning”). To support the contextual appropriateness of the adapted version, one experienced GFL instructor with over 5 years of teaching experience was invited to review the items. In addition, before the main data collection, seven third-year German-major students who did not participate in the final study completed the questionnaire to check item clarity and comprehensibility. Their feedback was used to refine item wording where necessary before the final version was administered. The adapted measure demonstrated strong reliability (Cronbach's alpha = 0.930) and structural validity (χ^2^/df = 366.792/246 < 3; CFI = 0.967 > 0.90; TLI = 0.963 > 0.90; SRMR = 0.038 < 0.08; RMSEA = 0.045 < 0.08), supporting its appropriateness for the GFL context.

#### 3.3.2 German academic achievement

German academic achievement serves as the dependent variable in this study. Scores from the PGG were used to measure German achievement. This standardized examination, administered annually in June by the German Subcommittee of the Foreign Language Teaching Advisory Board under the Ministry of Education, is designed for second-year German majors in mainland China and has a maximum score of 100. The PGG consists of five components: dictation (10%), listening comprehension (20%), reading comprehension (20%), grammar and vocabulary in context (35%), and written production (15%; Kong et al., 2014). These components primarily assess learners' receptive skills and form-focused language knowledge, together with controlled written expression. As a written and individually administered examination, the PGG places greater emphasis on text-based processing and linguistic accuracy, and does not directly assess spontaneous oral interaction or collaborative communicative performance. In the survey, students were asked to report their PGG scores by responding to the following question: “What was your score on the Prüfung für das Germanistik-Grundstudium? (Please enter a number, not a grade).” All responses were collected anonymously. Although the PGG itself is a standardized examination, the scores used in this study were obtained through participant self-report rather than official score records. Accordingly, the present study treats the variable as learners' reported performance on a standardized test, while recognizing that the use of self-reported scores may introduce recall error or other forms of reporting bias.

### 3.4 Data analyses

Prior to the main analyses, outliers were identified and processed using Excel, while missing values were uniformly coded as −999 in SPSS 27.0 for subsequent analysis. The reliability and structural validity of the four dimensions of the Social-Emotional Competence Questionnaire for German Learning were evaluated utilizing SPSS 27.0 and Mplus 8.0. Descriptive statistics and Pearson correlations were computed in SPSS 27.0, and structural equation modeling was conducted in Mplus 8.0.

Given the cross-sectional, self-report nature of the study, common method bias (CMB) was evaluated using Harman's single-factor test (Podsakoff et al., 2003). An exploratory factor analysis constraining all 24 items to a single factor yielded an explained variance of 38.90%, below the 50% threshold. A single-factor confirmatory model further confirmed poor fit (CFI = 0.571, TLI = 0.530, RMSEA = 0.158, and SRMR = 0.137), indicating that a single common factor could not adequately account for the data. These results suggest that CMB did not pose a serious threat to the validity of the findings.

Preliminary independent-samples *t*-tests were conducted to examine potential gender differences across the four SEC dimensions and German achievement scores. A significant gender difference emerged for self-management, with male students scoring higher than female students (*M* = 3.76, *SD* = 0.90 vs. *M* = 3.42, *SD* = 0.75), *t*_(245)_ = 2.81, *p* = 0.005, and *d* = 0.43. No significant gender differences were found for self-awareness, *t*_(245)_ = 1.26, *p* = 0.210, social awareness, *t*_(245)_ = 1.65, *p* = 0.101, social management, *t*_(245)_ = 0.03, *p* = 0.976, or German achievement, *t*_(245)_ = 0.87, *p* = 0.383. Although the effect size for self-management was small by L2 research standards (*d* = 0.43; Plonsky and Oswald, 2014), and given the pronounced gender imbalance in the sample (21.5% male, 78.5% female), gender was incorporated as an exogenous control variable in the structural equation model, with direct paths drawn from gender to all endogenous variables (e.g., Kline, 2023). None of the gender paths reached statistical significance, and the primary structural paths remained virtually unchanged after controlling for gender (Δβ < 0.01), confirming the robustness of the observed SEC-achievement relationships.

Regarding sample size adequacy, the present sample of 247 participants exceeds the *N* = 200 threshold commonly cited as a practical minimum for SEM with maximum likelihood estimation (Boomsma and Hoogland, 2001; Kline, 2023), and all standardized factor loadings were strong (range: 0.671–0.854), a condition associated with reduced sample size requirements (Wolf et al., 2013). Bootstrap resampling (*k* = 2,000) with bias-corrected confidence intervals was employed for all indirect effect estimates. The overall model fit indices (CFI = 0.968, TLI = 0.964, RMSEA = 0.042, and SRMR = 0.038) further support the stability of the solution (Hu and Bentler, 1999).

## 4 Results

### 4.1 Descriptive statistics

For analytical clarity, the mean scores for each of the four social-emotional competence dimensions were used to represent individual proficiency levels. Adopting Oxford's (1990) benchmarks, mean scores of 1.0–2.4 were classified as low, 2.5–3.4 as moderate, and 3.5–5.0 as high. As shown in Table 1, students demonstrated moderate-to-high scores across all four dimensions. Self-awareness (*M* = 3.99), social awareness (*M* = 3.75), and social management (*M* = 3.71) all reflected relatively high scores, whereas self-management (*M* = 3.49) was moderate. Correlation analyses showed that all variables were significantly positively correlated (*p* < 0.01). According to Cohen's (1988) criteria, correlation coefficients of 0.10, 0.30, and 0.50 represent small, medium, and large effect sizes, respectively. German achievement was strongly correlated with self-awareness (*r* = 0.56) and self-management (*r* = 0.50), and moderately correlated with social awareness (*r* = 0.37) and social management (*r* = 0.38).

**Table 1:** Descriptive statistics and correlations.

Variable	*M* (*SD*)	1	2	3	4	5	6
1. Self-awareness (1–5)	3.99 (0.72)	—					
2. Self-management (1–5)	3.49 (0.80)	0.41^**^	—				
3. Social awareness (1–5)	3.75 (0.80)	0.33^**^	0.50^**^	—			
4. Social management (1–5)	3.71 (0.77)	0.41^**^	0.36^**^	0.51^**^	—		
5. Total SEC scores (4–20)	14.95 (2.32)	0.70^**^	0.76^**^	0.78^**^	0.76^**^	—	
6. German achievement (0–100)	77.73 (9.63)	0.56^**^	0.50^**^	0.37^**^	0.38^**^	0.60^**^	—

M, mean; SD, standard deviation; SEC, social-emotional competence.

^**^p < 0.01 (two-tailed).

### 4.2 Mediation model analysis

#### 4.2.1 Model fit evaluation

To investigate the mechanisms through which SEC was associated with the participants' German academic achievement, a structural equation model was constructed to test the hypotheses that were illustrated in Figure 1. The model was estimated using maximum likelihood methods in Mplus 8.0. Confirmatory factor analysis was conducted to validate the measurement models for each latent variable. The results indicated that all four SEC dimensions demonstrated satisfactory construct validity, with all item factor loadings exceeding 0.60, suggesting that each subscale reliably captured its intended construct. Fit indices for the overall model indicated a good fit to the data (Hu and Bentler, 1999): χ^2^/df = 385.84/267 < 3, CFI = 0.97 > 0.95, TLI = 0.96 > 0.95, RMSEA = 0.04 < 0.06, and SRMR = 0.04 < 0.08.

In addition to model fit, the *R*^2^ values for the endogenous variables indicated that the model accounted for 44.4% of the variance in GFL achievement (*R*^2^ = 0.444, *SE* = 0.052, and *p* < 0.001), 36.7% of the variance in self-management (*R*^2^ = 0.367, *SE* = 0.057, and *p* < 0.001), and 40.9% of the variance in social management (*R*^2^ = 0.409, *SE* = 0.059, and *p* < 0.001). By Cohen's (1988) conventional benchmarks, all three values exceed the threshold for a large effect size (*R*^2^ > 0.26), indicating strong explanatory power of the hypothesized model.

#### 4.2.2 Path and mediation analysis

**Figure 2:**
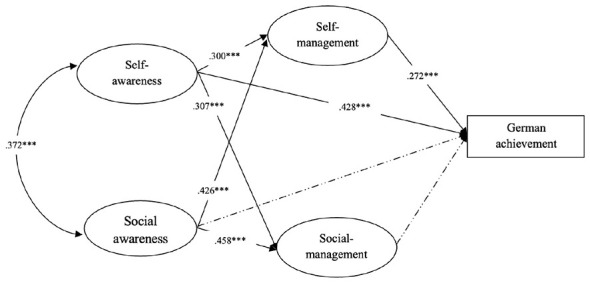
Structural model path estimates. Solid lines represent significant paths, and dotted lines represent non-significant paths. ***p < 0.001.

The results of the path analysis (see Table 2 and Figure 2) revealed the relationships among the latent variables in the model tested. Self-awareness had a significant positive effect on German academic achievement (β = 0.428, *p* < 0.05), as did self-management (β = 0.272, *p* < 0.05). Additionally, self-awareness significantly predicted self-management (β = 0.300, *p* < 0.05), and social awareness showed a significant positive association with self-management (β = 0.426, *p* < 0.05). Self-awareness also positively predicted social management (β = 0.307, *p* < 0.05), as did social awareness (β = 0.458, *p* < 0.05). However, neither social awareness nor social management showed a significant direct effect on German academic achievement.

**Table 2:** Path coefficients of the structural equation model.

Path	B	β	S.E.	C.R.	*p*
Self-awareness → German achievement	7.47	0.428	0.066	6.506	^***^
Self-management → German achievement	3.45	0.272	0.069	3.971	^***^
Social awareness → German achievement	0.69	0.046	0.078	0.582	0.561
Social management → German achievement	1.02	0.067	0.074	0.912	0.362
Self-awareness → Self-management	0.42	0.300	0.062	4.804	^***^
Social awareness → Self-management	0.51	0.426	0.063	6.805	^***^
Self-awareness → Social management	0.35	0.307	0.070	4.387	^***^
Social awareness → Social management	0.46	0.458	0.067	6.822	^***^

B, unstandardized path coefficient; β, standardized path coefficient; S.E., standard error; C.R., critical ratio.

^***^p < 0.001 (two-tailed).

To further examine the specific pathways through which the four dimensions of SEC relate to German academic achievement, a structural equation model was created with self-management and social management serving as mediators. A bias-corrected bootstrapping procedure employing 2,000 resamples was used to assess the significance of indirect effects. An effect was deemed to be significant if its 95% confidence interval did not include zero (Preacher and Hayes, 2008). Table 3 presents the standardized estimates for total, direct, and indirect effects, as well as the proportion of each effect in the structural equation model. The results indicated that self-awareness showed both a significant direct association with German achievement (β = 0.428) and a significant indirect effect through the two mediators (β = 0.102), with the 95% confidence interval excluding zero, indicating statistical significance. In contrast, the effect of social awareness on German achievement was only indirect (β = 0.147).

**Table 3:** Total, direct, and indirect effects with bootstrap confidence intervals.

Predictor	Effect type	Effect value	95% CI lower	95% CI upper	Proportion (%)
Self-awareness → German achievement	Total	0.530	0.416	0.634	/
	Direct	0.428	0.297	0.554	80.75
	Indirect	0.102	0.038	0.173	19.25
Social awareness → German achievement	Total	0.193	0.068	0.318	/
	Direct	0.046	−0.104	0.203	/
	Indirect	0.147	0.054	0.250	76.17

Effect values and confidence intervals are based on 2,000 bootstrap samples.

CI, confidence interval; Proportion, the percentage of the total effect.

To further investigate the significance of the mediation effects, the bootstrapping procedure with 2,000 resamples was performed to test the mediating paths in the structural equation model. As shown in Table 4, the 95% CI for Path 1 did not include zero, indicating that self-management significantly mediated the relationship between self-awareness and German achievement (indirect effect β = 0.082). Likewise, the 95% CI for Path 3 excluded zero, demonstrating a significant indirect effect of self-management between social awareness and German achievement (indirect effect β = 0.116). Together with the results in Table 3, these findings indicate that self-management fully mediates the association between social awareness and German achievement. In contrast, the 95% CIs for the mediation paths involving social management (Paths 2 and 4) were not significant. Thus, social management did not serve as a mediator between either self-awareness or social awareness and German achievement.

**Table 4:** Mediation effects: bootstrap test.

Path	Effect value	Proportion of indirect effect (%)	95% CI lower	95% CI upper
Path 1	0.082	80.39	0.039	0.141
Path 2	0.021	/	−0.024	0.070
Path 3	0.116	78.91	0.054	0.191
Path 4	0.031	/	−0.032	0.103

Effect values and confidence intervals are based on 2,000 bootstrap samples.

CI, confidence interval; Proportion, the proportion of the respective indirect pathway among total indirect effects.

Path 1: Self-awareness → Self-management → German achievement; Path 2: Self-awareness → Social management → German achievement; Path 3: Social awareness → Self-management → German achievement; Path 4: Social awareness → Social management → German achievement.

## 5 Discussion

The present study explored the relationship between social-emotional competence and academic achievement among Chinese students learning L3 German by utilizing structural equation modeling. The model revealed complex internal mechanisms by which the four dimensions of social-emotional competence showed differential associations with German learning outcomes. Our findings showed that the overall SEC of German-major undergraduates was moderately high. Among the four dimensions, self-awareness, social awareness, and social management were relatively high, whereas self-management was at a medium level. This profile suggests that students can accurately perceive their emotional states (e.g., enjoyment, anxiety, and boredom) during German learning, recognize the importance of a positive attitude for language learning, and understand their own strengths, weaknesses, and learning goals. However, students may still need support in translating self-awareness into intrinsic motivation, thereby strengthening self-management skills. The relatively high scores on social awareness and social management suggest that participants placed value on social activities (e.g., “My classmates and I collaborate in group work for German learning”) and social relationships (e.g., “I know from whom [e.g., siblings, parents, peers, and teachers] I can seek help when learning German”) in the context of learning German, and that they actively engaged in such activities.

Furthermore, social-emotional competence was significantly positively correlated with German academic achievement. Specifically, learners with higher social-emotional competence tended to demonstrate higher German achievement. This finding aligns with results from previous studies conducted on other languages (Durlak et al., 2011; Liu et al., 2022; Wu and Wang, 2017). Moreover, self-awareness showed the highest correlation with German achievement. This suggests that students who can accurately identify their emotions in German learning. This finding aligns with previous research showing that positive emotions in foreign language learning can positively predict language achievement, whereas negative emotions have a hampering effect. For instance, Dewaele and Alfawzan (2018) found that the facilitative effect of foreign language enjoyment on performance outweighed the negative impact of anxiety. Similarly, Li and Han (2022) reported that learners with higher levels of positive emotions tended to perform better in language learning. In the present study, all four dimensions of SEC were significantly inter-correlated, indicating that these dimensions are not independent of each other, but rather work together through internal mechanisms in their associations with German achievement.

Additionally, the results of the path analyses and mediation tests revealed the underlying mechanisms by which the four dimensions of SEC were associated with German academic achievement. As was illustrated in Figure 2, self-management served as a mediator between self-awareness and German achievement. As anticipated on the basis of control-value theory (Pekrun, 2006; see Section 3.1), this mediating pattern is consistent with the view that learners' appraisals of control and value in relation to academic tasks are associated with distinct achievement emotions, which in turn are systematically related to engagement, strategy use, and performance. This suggests that self-awareness alone may be insufficient for strong academic outcomes. Rather, the extent to which learners translate self-awareness into concrete regulatory behaviors, as captured by self-management, may represent a key mechanism through which awareness-related competences are associated with achievement in the GFL context.

Alongside this mediated pathway, self-awareness also showed a significant direct association with German achievement, indicating that its contribution operates through both direct and indirect routes. The core of self-awareness involves an individual's ability to recognize their own emotions, sense of self-efficacy, and clarity of meaning and purpose (Cefai and Cavioni, 2014). In the context of foreign language learning, this construct aligns with Garrett's (2010) concept of language attitude (cognitive, affective, and behavioral). Li and Li (2024) found that the more positive a learner's language attitude, the more likely they were to achieve positive results in foreign language learning; the findings of the present study somewhat corroborate these patterns.

Before examining the null findings for social awareness and social management, it is worth noting the significant path from social awareness to social management within the model. Students with higher social awareness demonstrated higher social management skills. Battistich et al. (2004) found that when the environment emphasizes sharing and mutual support, students are more likely to feel connected to their group and to participate in classroom activities, thereby improving their learning and behavior. This finding supports the path from social awareness to social management in our model: students with strong social awareness tended to actively engage in group collaboration in German learning.

However, neither social awareness nor social management showed a significant direct association with German achievement in the present study (social awareness: β = 0.046, *p* = 0.561; social management: β = 0.067, *p* = 0.362). These findings can be interpreted from three complementary perspectives.

The first concerns the nature of the PGG examination. As a written test comprising dictation, listening comprehension, reading comprehension, grammar and vocabulary, and written production, the PGG assesses individual, text-based language skills rather than collaborative or communicative performance. Social awareness and social management, as competencies most relevant to interpersonal interaction, may have limited effects in the PGG examination. Evidence from cross-national research suggests that the contribution of social-emotional skills to academic achievement varies systematically across skill types, with interpersonal competencies showing weaker links to test-based performance than intrapersonal, self-regulatory ones (Liu et al., 2025).

The second interpretation concerns the cultural context of Chinese higher education. China's collectivist cultural norms, including face concerns (*mianzi*) and the pronounced power distance between students and teachers (Hofstede, 2001), further shape how social competencies are enacted in classroom settings. Research has shown that Chinese foreign language learners are often reluctant to initiate classroom communication, fearing negative evaluation from both teachers and peers (Liu and Jackson, 2008). This reticence is not simply a matter of low social awareness. Peng and Woodrow (2010) likewise showed that classroom environment and learner beliefs were both implicated in the network of factors shaping willingness to communicate in EFL classrooms at Chinese universities. In these classrooms, students are often positioned as mentally rather than verbally active participants, and frequent voluntary speaking may risk being interpreted as inappropriate self-display (Peng, 2012). A socially aware student may therefore choose to internalize social insights as self-regulatory strategies instead of engaging in peer interaction. This cultural dynamic may help to explain why the advantage of social competences is mediated through self-management rather than through a direct association with achievement.

The third interpretation draws on sociocultural theory (Vygotsky, 1978), which was briefly discussed in Section 3.1. Sociocultural theory holds that human mental functioning is essentially a mediated process that is organized by cultural artifacts, activities, and concepts (Ratner, 2002). With respect to language learning, within this framework, learners develop language proficiency through engagement in culturally and historically situated environments, such as in family, peer group, and classroom activities (Melani et al., 2020). Social interaction is understood to facilitate the transition from inter-mental (shared between individuals) learning processes to intra-mental (internal to individuals) processes (Mitchell and Myles, 2004). The present findings are consistent with this view: Learners who reported stronger social awareness and greater engagement with peers also tended to show higher self-management scores, which in turn showed a significant positive association with German academic achievement. This suggests that socially available resources may have been internalized as individual regulatory strategies rather than expressed through direct behavioral pathways to performance. This interpretation is empirically supported by Zhang et al. (2026), who, in a Chinese university EFL context, also confirmed the social awareness → self-management pathway and a comparable indirect effect on language achievement through self-management. Notably, the accompanying null effect of social management on achievement has been replicated across secondary and university English learning contexts in China (Bai et al., 2024; Shen et al., 2024; Zhang et al., 2026), suggesting that both patterns reflect cross-linguistically stable features of SEC functioning in Chinese foreign language education rather than artifacts of the present design.

Taken together, these three interpretive perspectives suggest that the null direct effects of social awareness and social management are not simply an artifact of the measure. Instead, they reflect a theoretically coherent pattern specific to this institutional and cultural context. Rather than undermining sociocultural theory, these findings call for a more contextually sensitive application of sociocultural frameworks in L3 research: one that accounts for how examination-oriented learning ecologies and collectivist classroom norms may redirect the contribution of socially oriented competences from direct behavioral expression to internalized self-regulation.

Extending the work of Bai et al. (2024), our findings have identified a new path such that self-awareness showed a significant positive association with social management. Bai et al. (2024) suggested that the absence of a self-awareness → social management path in their adolescent sample might stem from the cognitive immaturity of younger learners, which makes it difficult for them to convert self-awareness (e.g., self-recognition) into social management skills (e.g., cooperative learning). In contrast, the university students in our sample are more cognitively mature than secondary students, and thus are more likely to be capable of translating self-awareness into management skills. University students also tend to exhibit greater learning autonomy and more diverse learning strategies in foreign language learning. Gao and Liu (2014) found that university-level Chinese-English learners could effectively use affective control strategies and metacognitive strategies to regulate their English learning. Kerr et al. (2006) similarly noted that emotional intelligence plays a crucial role in an individual's social effectiveness, and that those who manage their emotions well are often more willing to actively engage in social cooperation. Consistent with these findings, in our study, learners with high self-awareness also possessed high social management skills, enabling them to better participate in peer interactions and group collaborations in German learning.

Some consideration should also be given to the regional and institutional composition of the sample in the present study. The universities were distributed across geographically distinct regions of China and represented different tiers of the national higher education system. This range represents the contextual breadth and diversity across GFL at Chinese universities. At the same time, variation in institutional resources, instructional environments, and regional educational conditions may have introduced heterogeneity into the SEC-achievement relationship in ways that the current study was not designed to address. Whether and how these contextual factors moderate the pathways identified here remains an open question, and future research incorporating more purposive, stratified sampling designs would be better placed to examine it.

## 6 Limitations

The present study provides empirical evidence for significant association between SEC and L3 achievement among Chinese GFL learners, underscoring the value of integrating social-emotional learning within university language curricula. Nevertheless, several limitations warrant acknowledgment. Our participants were all third-year German GFL students, which does not capture the full range of learners in this context (e.g., from beginning students to advanced graduate students). Future research could adopt more diverse samples to examine how the associations between SEC and foreign language achievement may vary across developmental stages. Moreover, academic achievement was measured using a single standardized exam. Future research should aim to incorporate multiple measures of both SEC and academic achievement (e.g., classroom assessment, teacher ratings, and self- and peer-evaluation), ideally among more diverse samples and on a longitudinal scale. Comparative studies across languages and educational settings could further illuminate the generalizability and boundary conditions of the findings from the present study. Additionally, future work may wish to incorporate qualitative measures that could complement quantitative approaches by exploring how learners perceive and enact SEC in real-world language learning contexts.

The sample size may also be a limitation in the present study. Although the sample size of 247 participants exceeds commonly cited thresholds for SEM (Boomsma and Hoogland, 2001; Kline, 2023), and the strong factor loadings observed across all constructs (standardized range: 0.671–0.854) were associated with more favorable estimation conditions (Wolf et al., 2013), the relatively large number of free parameters in the model means that the parameter-to-sample ratio falls below conventional recommendations (Bentler and Chou, 1987). Replication with larger and more structurally diverse samples is therefore encouraged to confirm the stability of the reported estimates. A related concern pertains to the overrepresentation of “Double First-Class” universities in the present sample (52.38%). Students admitted to these universities are, by selection, among the higher-achieving end of the national GFL learner population, which may have introduced a ceiling effect on both SEC scores and PGG performance. Restricted variance in these variables could attenuate the observed path coefficients and limit the generalizability of the structural relationships to less selective institutional contexts. Future research employing purposive stratified sampling across institutional tiers would be better placed to examine whether the pathways identified here remain stable across the full range of Chinese GFL learners.

Finally, the measure of academic achievement may be considered a limitation. Although the PGG is a standardized and nationally recognized examination, the scores used in this study were self-reported by participants rather than verified against official records. Self-reported test scores may be affected by recall error, social desirability bias, or motivated misreporting (Kuncel et al., 2005; Mayer et al., 2007), which means that the achievement variable cannot be treated as fully objective in the present design. Such bias may have influenced the magnitude of the observed associations. Future research should consider gathering official scores/records or combining standardized examination data with additional achievement indicators.

## 7 Conclusion

The current study has systematically examined SEC and academic achievement among GFL learners at Chinese universities. Our findings showed that SEC was positively correlated with German learning outcomes, with the specific dimensions of self-awareness and self-management showing the highest associations, and social awareness and social management exhibiting moderate correlations. Additionally, we found that self-awareness showed significant positive associations with German achievement both directly and indirectly as mediated by self-management. Although social awareness did not show a significant direct association with German achievement, it showed a significant indirect association through self-management.

The current study offers three main contributions. Firstly, it extends the concept of SEC—widely discussed in education—into the scope of foreign language learning. By investigating how social-emotional competence relates to foreign language achievement, the findings from the present study broaden the perspective and scope of research in applied linguistics. Secondly, whereas prior studies have focused on L2 learners of English, our study reports on Chinese learners of German L3, expanding our knowledge of the role of SEC in learning LOTEs. Thirdly, earlier research had not examined the internal mechanisms by which SEC's sub-dimensions relate to academic outcomes. The current study addressed this gap by examining the interaction of four specific dimensions of SEC, shedding light on the complex mechanisms underlying the associations between SEC dimensions and L3 achievement. By mapping the pathways through which SEC dimensions are associated with academic outcomes, the present study offers a foundation for both theoretical refinement and practical innovation in L3 teaching and learning.

## Data Availability

The raw data supporting the conclusions of this article will be made available by the authors, without undue reservation.

## Appendix

### Social-Emotional Competence Questionnaire for German Language Learning

*Instructions*: Please indicate the extent to which you agree with each of the following statements about your experiences in learning German, using the following scale:

1, Strongly Disagree; 2, Disagree; 3, Neutral; 4, Agree; 5, Strongly Agree.

**Self-awareness**

1. I am aware of my feelings (e.g., enjoyment, anxiety, and boredom) when learning German.
2. I know my strengths and weaknesses in German learning.
3. I am clear about my goals in German learning and how to achieve them.
4. I understand the importance of maintaining a positive attitude toward German learning.
5. I realize that my success in learning German depends primarily on myself.

**Social awareness**

6. I believe that classmates should support each other in German learning.
7. I know from whom (e.g., siblings, parents, peers, and teachers) I can seek help when learning German.
8. I am aware of the resources my family provides for my German learning (e.g., tutoring, books, courses).
9. I am aware of the resources available from my university for German learning (e.g., library, German club, overseas exchange programs).
10. I believe that group activities can better facilitate German learning.
11. My classmates' successful experiences in German learning offer me new insights.
12. I am aware that my contributions to group activities are important in German learning.

**Self-management**

13. I can manage my emotions (e.g., happiness, anxiety, and boredom) during German learning.
14. I can solve problems independently in German learning.
15. I can find effective methods for learning German.
16. I can set and achieve goals in German learning.
17. I am able to stay focused when learning German.
18. I am able to maintain a positive attitude toward German learning.
19. I am able to cope with challenges in German learning.

**Social management**

20. My classmates and I help each other in learning German.
21. My classmates and I collaborate in group work for German learning.
22. My classmates and I discuss issues related to German learning.
23. I seek advice from others (e.g., siblings, parents, peers, and teachers) in my German learning.
24. I try to figure out why some classmates are successful in learning German.
